# 循环肿瘤DNA在非小细胞肺癌诊疗中的研究进展

**DOI:** 10.3779/j.issn.1009-3419.2022.102.35

**Published:** 2022-09-20

**Authors:** 思雨 雷, 燕 王

**Affiliations:** 100021 北京，国家癌症中心/国家肿瘤临床医学研究中心/中国医学科学院北京协和医学院肿瘤医院肿瘤内科 Department of Medical Oncology, National Cancer Center/National Clinical Research Center for Cancer/Cancer Hospital, Chinese Academy of Medical Sciences and Peking Union Medical College, Beijing 100021, China

**Keywords:** 肺肿瘤, 循环肿瘤DNA, 研究进展, Lung neoplasms, Circulating tumor DNA, Research progress

## Abstract

随着恶性肿瘤精准化诊疗理念的不断深入，在从微观视角探索肿瘤发生发展的过程中涌现了许多对临床决策有价值的信息载体，循环肿瘤DNA（circulating tumor DNA, ctDNA）就是其中之一。在肿瘤发展的不同阶段，ctDNA展现出了包括评估疗效、预测预后、监测复发等在内的多种诊疗价值。本文主要阐述ctDNA在非小细胞肺癌临床诊疗不同时期的应用研究进展。

## Abstract (en)

With the concept of "Precision Medicine" in malignant tumors popularized, many substances carrying valuable clinical information have emerged in the process of exploring the occurrence and development of tumors from a microscopic perspective. Circulating tumor DNA (ctDNA) is one of them. In various clinical stages of cancer, ctDNA exhibits rich diagnostic values including demonstrating the efficacy of treatment, predicting prognosis, and monitoring disease recurrence. This article mainly describes the application and research progress of ctDNA in different stages of clinical diagnosis and treatment of non-small cell lung cancer.

## 1 循环游离DNA和循环肿瘤DNA的定义、来源和特点

### 1.1 循环游离DNA

循环游离DNA（circulating free DNA, cfDNA），也称无细胞DNA，是细胞凋亡、坏死或主动分泌至血液中的脱氧核糖核酸分子^[1, 2]^，大部分的cfDNA来源于造血细胞^[3]^，长度大约为167 bp，其半衰期在16 min-2.5 h之间^[4]^。健康人血液中cfDNA的水平在1 ng/mL-10 ng/mL之间，并在一些生理状态下会有一定范围的波动^[5, 6]^。肿瘤患者的cfDNA平均水平比正常人高^[7]^，由于血液中来自正常细胞的种系DNA水平基本保持不变，肿瘤细胞会释放其DNA至血液循环中，故而异常升高的cfDNA水平可能与肿瘤负荷相关^[8]^。循环肿瘤DNA（circulating tumor DNA, ctDNA）就是cfDNA中部分携带有肿瘤特异性基因变异的源自肿瘤细胞的DNA。

### 1.2 ctDNA

ctDNA片段长度在134 bp-144 bp之间，其血浆浓度与肿瘤大小和分期密切相关^[9, 10]^。非小细胞肺癌（non-small cell lung cancer, NSCLC）的ctDNA半衰期为35 min^[10]^，由于其半衰期较短，因此理论上血浆ctDNA检测可以“实时”反映体内肿瘤负荷。在实际临床应用中，ctDNA的检出受到一些因素的影响。一类因素涉及肿瘤自身特性。Abbosh等^[9]^将NSCLC患者血浆中所有克隆性单核苷酸突变（single-nucleotide variants, SNVs）的平均等位基因变异频率（variant allele frequency, VAF）与计算机断层扫描（computed tomography, CT）测量的肿瘤体积关联后发现，肿瘤体积与平均VAF呈正相关，说明肿瘤体积更大，ctDNA水平更高。此外，肿瘤分期晚、坏死程度高、病理类型为非腺癌、增殖能力强、淋巴管和血管浸润更严重也和更多的ctDNA释放相关^[11]^。一类因素有关检测技术方法，术后ctDNA检测的特异性和灵敏度在不同研究间差异较大，特异性在71%-100%之间，灵敏度在36%-100%之间^[4]^。这些差异除了来自检测技术以外，还与ctDNA阳性的定义不同有关。有研究^[12]^将血浆中存在一个或多个配对肿瘤组织中的突变定义为阳性，有研究^[13]^把血浆检测到的突变中有超过5%来自于肿瘤组织特异性突变的情形判定为阳性，有研究则^[9]^认为检测到≥2个SNVs即为阳性。不同的阳性判定方式一定程度上限制了研究之间的可比性，为了ctDNA能更好地发挥其临床作用，需要对其阳性判定方法进行规范和统一。

## 2 ctDNA在NSCLC不同诊疗时期中的研究进展

在NSCLC的不同诊疗时期，ctDNA的应用价值有所不同。在疾病筛查阶段，ctDNA被寄希望于成为一种无创、无诱发第二原发肿瘤风险、能够通过更加灵敏地动态监测过程实现肿瘤早诊目标的检测手段^[14]^。在疾病得到根治性治疗后，ctDNA是治疗后微小残留病灶（minimal residual disease, MRD）的一种分子表现形式，可用于筛选术后辅助治疗最佳获益人群，更加灵敏地发现疾病复发，对患者进行预后分层等。在疾病进展期，ctDNA可以在无法获取肿瘤组织时作为替代方法揭示肿瘤分子特点^[15]^，也可以作为组织活检的补充，发现肿瘤的分子异质性。动态监测ctDNA在预测和评估靶向治疗^[16]^以及免疫治疗疗效^[17]^、筛选免疫治疗长期获益人群^[18]^、早期发现疾病进展、判断肿瘤克隆进化模式以及指导治疗决策等诸多方面发挥着重要作用^[9]^。

### 2.1 ctDNA在新辅助治疗中的研究进展

NADIM研究^[19]^探究了治疗前（基线）ctDNA与预后的关系。这项单臂的Ⅱ期临床研究旨在探索术前应用纳武利尤单抗联合化疗治疗ⅢA期不携带表皮生长因子受体（epidermal growth factor receptor, *EGFR*）或间变性淋巴瘤激酶（anaplastic lymphoma kinase, *ALK*）基因变异NSCLC的疗效，主要研究终点是24个月无进展生存（progression-free survival, PFS）率。研究共入组46例患者，24个月PFS率为77.1%，病理完全缓解（pathological complete response, pCR）率达到63%。研究者进一步探索了ctDNA作为新辅助免疫联合化疗预后标志物的应用价值。研究者应用Oncomine Pan-Cancer Cell-Free Assay对43例患者进行ctDNA检测，其中30例患者（69.8%）在治疗前血液标本中检测到ctDNA，平均每例患者检测到2个突变，当研究者将ctDNA水平定义为所有等位基因突变频率（mutant allele frequency, MAF）之和时，ctDNA水平 < 1%者较≥1%者有更长的PFS和总生存期（overall survival, OS），风险比（hazard ratio, HR）分别为0.22（*P*=0.016）和0.04（*P*=0.008）；若把这个分界值设为2%，也可得到相似的结论，其中PFS和OS的HR分别为0.19（*P*=0.009）和0.10（*P*=0.011）。当研究者将ctDNA水平定义为最大MAF时，最大MAF < 1%者有更长的PFS和OS，HR分别为0.26（*P*=0.033）和0.05（*P*=0.012）。相比之下，根据实体肿瘤疗效评价标准（Response Evaluation Criteriain in Solid Tumors, RECIST）评估的临床反应与PFS或OS之间没有显著关联^[20]^。以上结果提示，在新辅助免疫联合化疗的背景下，治疗前ctDNA水平似乎比常规影像学手段在评估预后方面有更高的价值，未来能否将其作为新辅助免疫联合化疗的预后标志物以及如何界定其cut-off值还需更多研究进行探索和验证。

CheckMate-816研究^[21]^探索了新辅助免疫联合化疗治疗期间ctDNA动态变化与肿瘤病理缓解和疾病预后的关系。这项Ⅲ期临床研究旨在探究IB期-ⅢA期NSCLC术前使用纳武利尤单抗联合化疗对比单纯化疗的pCR率和无事件生存期（event-free survival, EFS）。研究结果显示，免疫联合化疗组显著提高了pCR率，延长了EFS。该研究的探索性部分使用ArcherDx技术平台分析新辅助治疗过程中ctDNA水平变化，将ctDNA水平在新辅助治疗的第1个周期（C1D1）可测而第3个周期（C3D1）不可测定义为ctDNA清除。结果显示，有87例患者在C1D1可检测到ctDNA，其中纳武利尤单抗联合化疗组43例，化疗组44例，两组分别有24例（56%）和15例（35%）患者达到ctDNA清除（*P*=0.042）。两组达到ctDNA清除者的pCR率均较未达到者更高，EFS均呈现更长的趋势，此外，免疫联合化疗组达到pCR者EFS更长（HR=0.13, 95%CI: 0.05-0.37），提示新辅助治疗期间出现ctDNA清除可能代表着更佳的治疗应答，是正向预后因素，是否可以将ctDNA清除作为预后的替代指标，或用于指导新辅助治疗方案规划还有待进一步探究。

LCMC3研究^[22]^探索了新辅助免疫治疗前后ctDNA变化和疗效的关系。这项Ⅱ期临床研究结果显示对于IB期至部分ⅢB期NSCLC患者，术前应用2个周期阿替利珠单抗后主要病理缓解率（major pathological response, MPR）为21%。研究进一步对阿替利珠单抗治疗前后及手术后ctDNA进行监测，使用AVENIO ctDNA检测设备，并采取了肿瘤先验（tumor-informed）和胚系矫正（germline correction）的方法以提高检测准确性和灵敏度。ctDNA阳性指研究者定义的ctDNA检测指数 < 0.05。结果显示，基线ctDNA阳性率为72%，阿替利珠单抗治疗后ctDNA阳性率下降至56%，术后进一步下降至10%，中位ctDNA水平也呈类似的阶梯式下降。达到MPR者的中位ctDNA下降幅度比未达到者更大（*P* < 0.001）。该研究说明新辅助免疫治疗前后ctDNA的下降幅度与病理反应和影像学上肿瘤缩小程度有关。

上述研究表明，在新辅助治疗阶段，ctDNA的基线水平和其在治疗期间的动态变化与肿瘤病理缓解程度和预后相关，这种相关性在未来或可成为筛选新辅助治疗获益人群、规划新辅助治疗方案以及监测治疗效果的生物标志物。随着未来更多新辅助治疗临床试验的结果陆续成熟，ctDNA在这个阶段所扮演的角色将会进一步明确。

### 2.2 ctDNA在根治性治疗后的应用探索

2021年Qiu等^[12]^在*Nature Communications*上发表的文章阐释了ctDNA检测在预测NSCLC辅助治疗获益和术后复发风险方面的应用价值。研究者采用肿瘤先验的方法，用预先设计好的肿瘤基因下一代测序面板（next generation sequencing panel）对配对的肿瘤样本和血浆样本进行分析，将ctDNA阳性定义为在血浆样本中可检测到一种或多种配对肿瘤样本中存在的突变。对85例术前检测到体细胞突变且术后可获得血浆样本的患者进行ctDNA检测，其中18例患者术后ctDNA阳性，相比于术后ctDNA阴性的患者，这些阳性患者的无复发生存期（recurrence free survival, RFS）显著缩短，且无论其是否接受术后辅助治疗，术后ctDNA阳性均与更差的RFS显著相关。同年，Xia等^[23]^在*Clinical Cancer Research*上发表的“LUNGCA”研究得出了类似的结论：术后ctDNA（MRD）阳性强烈预示疾病复发（HR=11.1, *P* < 0.001），且其对预测效力的贡献超越了包括肿瘤原发灶-淋巴结-转移（tumor-node-metastasis, TNM）分期、肿瘤大小、组织学类型、年龄和辅助治疗情况在内的临床病理学指标贡献之和。此项研究还发现术前ctDNA阳性者的RFS更短（HR=4.2, *P* < 0.001），且这种关联在腺癌中显著（HR=6.0, 95%CI: 3.4-10.6, *P* < 0.001），在鳞癌中并未呈现出来（HR=2.4, 95%CI: 0.5-10.7, *P*=0.241），这可能与鳞癌的ctDNA比腺癌更易“脱落”至循环中而被检测到有关，事实上，此项研究中，鳞癌术前ctDNA的阳性率（74.4%）的确高于腺癌（11.8%），这个结果也与前述研究一致。

除了探索术后ctDNA状态与预后的关系之外，Chen等^[13]^还报道了用ctDNA状态来分析术后辅助治疗获益的可行性。研究者发现，在ctDNA阳性患者中，接受辅助治疗与更好的RFS相关，而在ctDNA阴性患者中，是否接受辅助治疗对RFS无影响。与Chen等^[13]^的研究稍有不同的是，Xia等^[23]^发现，在术后ctDNA阴性者中，接受辅助治疗患者的RFS显著低于未接受辅助治疗者（HR=3.1, *P* < 0.001），进一步研究发现，即使术后接受过辅助治疗，若辅助治疗后ctDNA阳性，则复发风险较辅助治疗后ctDNA阴性者高。相似的结论在结直肠癌术后ctDNA研究中也有阐述^[24]^，更重要的是，研究者还发现术后辅助治疗期间ctDNA的动态变化可以反映辅助治疗的效果，并提出将连续ctDNA监测作为辅助治疗疗效监测的标志物。另一项大型结直肠癌术后辅助治疗的随机对照研究^[25]^发现更长的术后辅助治疗期可能通过增加残留疾病清除率来降低ctDNA对预后的影响，且这种影响对于ctDNA阳性者有更显著的趋势。遗憾的是，该研究未对辅助治疗期间的ctDNA情况进行进一步动态监测，因此无法评估ctDNA下降或清除情况对疾病缓解的影响。这些研究结果对NSCLC术后ctDNA应用探索有所启发，但是否能够得出类似的结论还需要进一步验证。

在术后辅助免疫治疗方面，IMpower010研究的成功使得辅助阿替利珠单抗治疗被美国国立综合癌症网络（National Comprehensive Cancer Network, NCCN）指南推荐用于Ⅱ期-ⅢA期程序性死亡配体1（programmed cell death ligand 1, PD-L1）≥1%（SP263）NSCLC术后辅助化疗后的患者。探索性研究发现，术后辅助治疗前ctDNA阳性者较阴性者无疾病生存期（disease-free survival, DFS）更短。在ctDNA阳性者中，阿替利珠单抗较最佳支持治疗能显著改善DFS（HR=0.61, 95%CI: 0.39-0.94），且这种改善更多地体现在PD-L1≥1%的人群中。在ctDNA阴性者中，两组的中位DFS均未达到，阿替利珠单抗似乎有获益趋势（HR=0.72, 95%CI: 0.52-1.00）。同样地，该研究并未在辅助治疗过程中监测ctDNA变化情况。

包括ADAURA研究^[26]^在内的一系列术后辅助靶向治疗研究使得表皮生长因子酪氨酸激酶抑制剂（epidermal growth factor receptor-tyrosine kinase inhibitors, EGFR-TKIs）成为IB期-Ⅲ期*EGFR*敏感突变患者术后辅助治疗方式之一。Liu等^[27]^发现并非所有*EFGR*敏感突变患者都能从术后辅助靶向治疗中获益，且辅助靶向治疗往往需要口服2年-3年靶向药，对于个体来说，获益是否能与风险相平衡是重要的考量因素。ctDNA能否筛选出此类患者中的最佳获益人群，在术后辅助靶向治疗中，若达到ctDNA清除，是否可以停用靶向药物，代以定期检测ctDNA和影像学来监测复发，从而使患者获得更好的生活质量？期待未来能有相关研究回答这个问题。

对于不可手术的患者，Moding等^[28]^探索了根治性同步放化疗（concurrent radiochemotherapy, CRT）后免疫巩固治疗期间ctDNA的预后价值。类似于术后，ctDNA对CRT后疾病进展的风险具有高度预测性，可以提前4.1个月预判影像学进展。开始免疫巩固治疗后仍可检测到ctDNA高度提示预后不良，而CRT后ctDNA阴性的患者则可能不会从免疫巩固治疗中获益。

上述研究带来的启示在于，术后辅助治疗应在更精准的获益人群分层上进行，并且需要更早期且直观的辅助治疗疗效监测方法，以适应性调整治疗强度，从而在最大化获益的同时平衡治疗相关风险的增加。可以预见的是，在未来的临床实践中，ctDNA检测将会是制定更加精准和个体化的早中期患者根治性治疗前后临床决策的重要参考依据。

基于这样的理念，一些ctDNA指导根治性治疗后患者管理的临床研究正在开展。MERMAID-2研究^[29]^对Ⅱ期-Ⅲ期NSCLC根治性治疗后的患者进行MRD动态监测，将MRD阳性且无影像学进展证据的人群随机分为度伐利尤单抗组和安慰剂组，治疗2年，主要研究终点为PD-L1肿瘤细胞（tumor cell, TC）≥1%患者的DFS。NCT04585477研究^[30]^对Ⅰ期-Ⅲ期根治性手术或放疗后的患者进行ctDNA检测，阳性者予以12个周期度伐利尤单抗治疗，并在治疗2个周期后再次进行ctDNA检测以评估2个周期的治疗对ctDNA的影响，主要研究终点为2个周期治疗后ctDNA水平下降≥3倍的人数。NCT04585490研究^[31]^对同步放化疗后ctDNA阳性的受试者进行4个周期化疗联合度伐利尤单抗治疗序贯度伐利尤单抗至1年，对于ctDNA阴性者使用度伐利尤单抗治疗1年，主要研究终点是ctDNA阳性者4个周期化疗联合度伐利尤单抗后ctDNA水平降低≥3倍的人数。期待这些研究的结果能够就上述设想给出答案。

### 2.3 ctDNA在晚期NSCLC中的研究进展

在肺癌的分子检测中，能获取肿瘤组织并直接对其进行二代测序固然应当被首先考虑，但临床上不难见到一些难以获取或难以取到足够量肿瘤组织以满足检测要求的情况，在一些经过多程治疗之后或体能状态较差无法耐受有创检测的患者中尤其多见。在这些情况下，ctDNA检测或许是更加合适的选择^[18]^。此外，肿瘤产生耐药性的过程往往与分子克隆进化相关，无论是对于临床诊疗还是科研探索，多次动态监测和跟踪分子克隆变化是揭示耐药机制、指导临床诊疗和科研方向的关键步骤，多次活检取材往往面临着伦理性和可行性的双重挑战，包括ctDNA检测在内的液体活检方法是一个理想的解决方案^[9, 32-34]^。

ctDNA能反映预后。Song等^[35]^探索了带有驱动基因突变的晚期NSCLC患者在各种治疗期间ctDNA的变化与生存的关系，发现较高的基线ctDNA含量（最大等位基因突变频率乘以cfDNA总量）与更差的OS相关。在治疗过程中，至少出现过一次ctDNA清除（用168基因的panel检测不到突变）的患者较ctDNA持续阳性者中位PFS和OS显著延长。在接受化疗的患者中，ctDNA能够预测PFS，但不能预测OS，因此，研究者认为ctDNA是监测实时疗效的较好生物标志物。此外，不同的ctDNA清除模式有不同的预后意义。相比于ctDNA未清除者，全部ctDNA清除者有生存获益，但仅驱动基因的清除并没有生存获益。研究者进一步探索了接受奥希替尼治疗者的ctDNA特征^[36]^，发现更高的基线VAF、合并*TP53*共突变、合并细胞周期相关基因共突变或合并拷贝数扩增是OS负性预测因素。在治疗2个周期后ctDNA清零与更好的PFS和OS有关。研究者将“分子进展”定义为出现新的突变或原有突变丰度增加，发现分子进展可以比影像学检查提前中位2.5个月预测进展的发生。

ctDNA鉴别免疫治疗假进展。已有文献报道ctDNA的变化模式可以鉴别恶性黑色素瘤免疫治疗中的假进展^[37]^，肺癌中出现免疫治疗假进展的比例虽不高，仅占0.6%-5.8%^[38]^，但是肺癌总体患病负担大，将这部分获益人群识别出来不仅有助于精准治疗的实施，还可以通过分析和总结此类人群的特点，形成对免疫治疗作用机制和模式更加深入的理解。Guibert等^[39]^检测了*KRAS*突变肺腺癌免疫治疗发生假进展的2例患者和真进展的1例患者的动态ctDNA，发现2例假进展患者基线ctDNA水平较高，并在2个周期治疗后显著下降，虽然2例患者在2个周期评价疗效时都出现了疑似疾病进展的影像学表现，但是继续免疫治疗后均获得了持久的临床获益。而另一例患者接受免疫治疗2个周期后ctDNA较基线上升超过10倍，并在治疗4个周期后影像学确认为疾病进展。以上结果说明ctDNA可能在准确评估免疫治疗假进展、早期反映免疫治疗疗效方面有更多应用前景。

ctDNA可以反映免疫治疗疗效和预后。Zhang等^[40]^报道了迄今为止样本量最大的与免疫治疗疗效相关的ctDNA动态变化数据。这项研究通过整合3项临床试验结果分析了来自16个瘤种的978例治疗前ctDNA水平和171例治疗中ctDNA水平数据。研究发现，治疗前ctDNA VAF与OS显著负相关，且这种相关性，在调整了美国东部肿瘤协作组（Eastern Cooperative Oncology Group, ECOG）体能状态评分、基线肝转移状态、基线淋巴结转移状态、吸烟状况、肿瘤负荷和肿瘤PD-L1等预后相关因素后仍保持其显著性，提示治疗前ctDNA可以稳健地预测预后。另一方面，治疗过程中ctDNA的水平和VAF的降低程度可预测免疫治疗的获益。为了进一步量化这种动态变化，研究者通过整合治疗前VAF和治疗中VAF的信息构建了一个公式，并将其简化为治疗中VAF与治疗前VAF的比值，结合公式，以50%作为cut-off值，比值< 0.5的为“分子反应者”， > 0.5的“无反应者”，并证实了公式与RECIST反应的相关性。类似地，Goldberg等^[41]^对接受免疫治疗NSCLC患者基线血标本中ctDNA VAF最高的突变进行持续监测，定义ctDNA VAF下降超过50%为“ctDNA反应”，结果发现，ctDNA反应与影像学反应保持高度一致，且ctDNA出现反应的时间较影像学反应出现时间提前42.5天，ctDNA反应者较不反应者中位治疗时间更长。

ctDNA可以预测免疫治疗长期获益后的进展风险。Hellmann等^[42]^分析了从免疫治疗中长期获益（PFS > 12个月）的晚期NSCLC患者中ctDNA对疾病进展的预测作用。研究者在免疫治疗开始后≥6个月收集血浆样本，中位采血时间为26.7个月，发现4例ctDNA阳性患者全部进展，27例ctDNA阴性患者中25例未进展，这些ctDNA阴性患者的中位PFS显著长于阳性者。检测或未检测到ctDNA的患者的体内残余肿瘤负荷和转移数目相似，因此排除了由于体内可见肿瘤负荷差异所致ctDNA检出率的不同。

## 3 总结

许多探索性研究已经证实ctDNA检测及其动力学评估可以在NSCLC治疗的各个时期评估治疗反应，预测预后，未来的大规模临床研究应该探究ctDNA指导下的分层干预是否能带来更多临床获益以及包括治疗前、治疗早期、治疗过程中以及进展期等不同临床情境下ctDNA诊断界值的界定，从而为更恰当和适时的临床干预提供可靠的依据。此外，ctDNA检测技术朝着稳定、精准、高效、低成本的方向不断发展是取得这些进步的关键。
